# Migration of germline progenitor cells is directed by sphingosine-1-phosphate signalling in a basal chordate

**DOI:** 10.1038/ncomms9565

**Published:** 2015-10-12

**Authors:** Susannah H. Kassmer, Delany Rodriguez, Adam D. Langenbacher, Connor Bui, Anthony W. De Tomaso

**Affiliations:** 1 Neuroscience Research Institute, University of California, Santa Barbara, California 93106, USA; 2 Department of Molecular, Cellular and Developmental Biology, University of California, Santa Barbara, California 93106, USA

## Abstract

The colonial ascidian *Botryllus schlosseri* continuously regenerates entire bodies in an asexual budding process. The germ line of the newly developing bodies is derived from migrating germ cell precursors, but the signals governing this homing process are unknown. Here we show that germ cell precursors can be prospectively isolated based on expression of aldehyde dehydrogenase and integrin alpha-6, and that these cells express germ cell markers such as *vasa*, *pumilio* and *piwi*, as well as *sphingosine-1-phosphate receptor*. *In vitro*, sphingosine-1-phosphate (S1P) stimulates migration of germ cells, which depends on integrin alpha-6 activity. *In vivo*, S1P signalling is essential for homing of germ cells to newly developing bodies. S1P is generated by sphingosine kinase in the developing germ cell niche and degraded by lipid phosphate phosphatase in somatic tissues. These results demonstrate a previously unknown role of the S1P signalling pathway in germ cell migration in the ascidian *Botryllus schlosseri*.

## Abstract (web summary)

The regulation of germ cell migration in the colonial ascidian *Botryllus schlosseri* is poorly understood. In this chordate, Kassmer *et al*. identify sphingosine-1-phosphate as regulating germ cell migration *in vitro* and homing of cells to newly developing bodies in live organisms.

## Graphical abstract

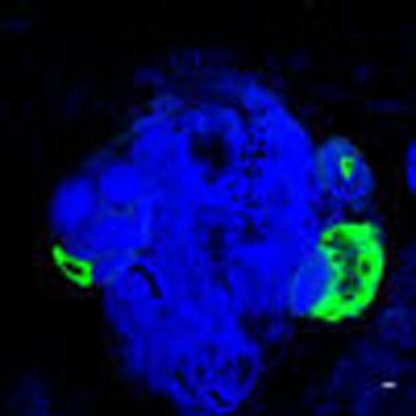

In most organisms, germ cells are specified early in development, and must migrate through and along various somatic tissues to reach the somatic niche of the gonad. In contrast to somatic tissues, which cease to exist when an organism dies, germ cells link successive generations together, and are, in that sense, immortal (reviewed in ref. 1). Ascidians are marine invertebrate chordates that straddle the evolutionary divide between invertebrates and vertebrates. Embryogenesis results in a swimming tadpole larva with characteristic chordate features that later undergoes metamorphosis into a sessile invertebrate adult. Colonial ascidian species increase in size via an asexual budding process, during which their entire bodies, including all somatic and germline tissues, are regenerated *de novo*. Colonies of *B. schlosseri* asexually reproduce every week, within a 2- to 3-year lifespan, providing a unique model to study asexual reproduction and regeneration^2^. Individuals within the colony are interconnected by a shared, extracorporeal vasculature, and are embedded in an extracellular matrix known as the tunic. Two generations of newly developing bodies, termed primary and secondary buds (Fig. 1a) grow from the adult bodies, known as zooids. Secondary buds begin as small protrusions of the body wall of primary buds, and proceed to form a closed vesicle, followed by invaginations and tissue differentiation, completing development into the adult form (Fig. 1a). The source of the germ line in each asexually derived generation is a population of migratory germ cell precursors, which migrate to new germline niches within the secondary bud at the double vesicle stage^3^ (Fig. 1a). These germ cell precursors then develop into functional gonads as the primary bud matures into an adult zooid^4^.

To date, germline stem cell (GSC) biology has been studied in a limited number of model organisms, and most of our knowledge comes from studies in flies and nematodes, which are phylogenetically very distant from vertebrates. In these species, germ cell fate is specified by a maternally synthesized germ plasm, whereas in mammals and many other chordates, germ cells form at later stages of development by inductive signals from neighbouring tissues^1^. Despite these large phylogenetic distances and differences in the mechanisms of germ cell specification, the genes that are critical for specifying and maintaining germ cell fate are highly conserved across phyla. Examples include *vasa*, *nanos, pumilio* and *piwi*, which are not only required for early germ cell specification, but throughout germline development in most organisms^5^. *Vasa* encodes an ATP-dependent RNA-helicase, and it is expressed by germ cells and primordial germ cells in most phyla studied to date^6^,^7^. *Vasa* is therefore a reliable marker for primordial germ cells in all animals. Germ cell migration has been studied in flies, zebrafish and mice, and although there are important differences in the underling mechanisms, several shared principles exist. For example, signalling from G-protein–coupled receptors appears to be essential for the directed migration of germ cells. Also, lipid signalling pathways play important roles in germ cell migration in several model organisms (reviewed in ref. 8). However, the specific receptors and signalling pathways can differ greatly between species. In *Drosophila*, directional migration of germ cells is guided by signalling from the receptor Tre-1, a G-protein–coupled receptor of the rhodopsin family, whereas in zebrafish and mice, CXCR4-signalling mediates the chemotactic response of germ cells. Therefore, to gain a deeper understanding of the mechanisms regulating germ cell migration and their evolution, it is essential to study these mechanisms across different phyla. Here, we aim to identify the pathways governing germ cell migration in *B. schlosseri*, an invertebrate chordate.

Sphingosine-1-phosphate (S1P) is a bioactive lipid-signalling molecule that regulates multiple essential cellular processes, including cell growth and survival, as well as cell motility and trafficking. Increased S1P production has been implicated in various pathophysiological processes, such as cancer, allergy and autoimmune diseases^9^. Studies in *Drosophila* have suggested a role for phospholipid signalling in regulating germ cell migration and survival^10^, but a role of S1P in germ cell migration has not been demonstrated in any species to date. S1P is generated by phosphorylation of sphingosine by sphingosine kinase 1 (Sphk1) at the inner leaflet of the plasma membrane^9^,^11^. This leads to spatially restricted formation of S1P that can be exported out of cells by ABC transporter family members. S1P can then bind to its receptor, S1P receptor type 1 (S1pr1), on the same or neighbouring cells to stimulate G-protein-regulated signalling pathways. Thus, intracellularly generated S1P can signal ‘inside-out’ through its cell surface receptors in an autocrine or paracrine manner^9^,^11^. S1P levels are tightly regulated by the balance between synthesis by Sphk1, reversible conversion to sphingosine by specific S1P phosphatases (Spp1 and Spp2) and other lipid phosphate phosphatases (Lpp’s), and irreversible degradation by S1P lyase^9^,^11^.

Previous studies have demonstrated that in *Botryllus*, a population of cells enriched for germ cell marker genes and GSC function can be prospectively isolated based on expression of aldehyde dehydrogenases (ALDH)^12^, although only 1 out of 50 ALDH-positive cells functionally reconstitutes the germ line of a recipient. Here, we develop a novel method of prospective isolation of a highly enriched population of germ cell precursors, based on expression of ALDH and integrin alpha-6. We show that integrin alpha-6 (Ia6) and S1pr1 are expressed on migrating *vasa*-positive germ cells, and that ALDH/Ia6-positive cells respond to S1P with increased migratory activity, whereas inhibition of S1P signalling disrupts homing of *vasa*-positive germ cells *in vivo*. We have identified S1P as a crucial chemoattractant-signalling pathway directing migration of germ cells in an invertebrate chordate.

## Results

### *Vasa*-positive germ cells express *integrin alpha-6* and *s1pr1*

Because of the established roles of S1P in migration of immune cells and haematopoietic stem cells^11^,^13^ in humans and mice, we aimed to analyse the expression of S1P receptor genes in germ cells from *Botryllus.* We performed a BLAST search, and found two S1P receptor genes in the *Botryllus* genome, which share sequence homology with the vertebrate genes *s1pr1* (28%, identity, *E*-value=1e-30) and *s1pr4* (35% identity, *E*-value=0.005).

Integrin alpha-6 is a marker on the surface of spermatogonial stem cells^14^ and embryonic stem cells^15^. We performed a BLAST search of non-redundant protein sequences, and found a *Botryllus* homologue of *integrin alpha-6* (34% identity, *E*-value=1e-166).

**Figure 1:**
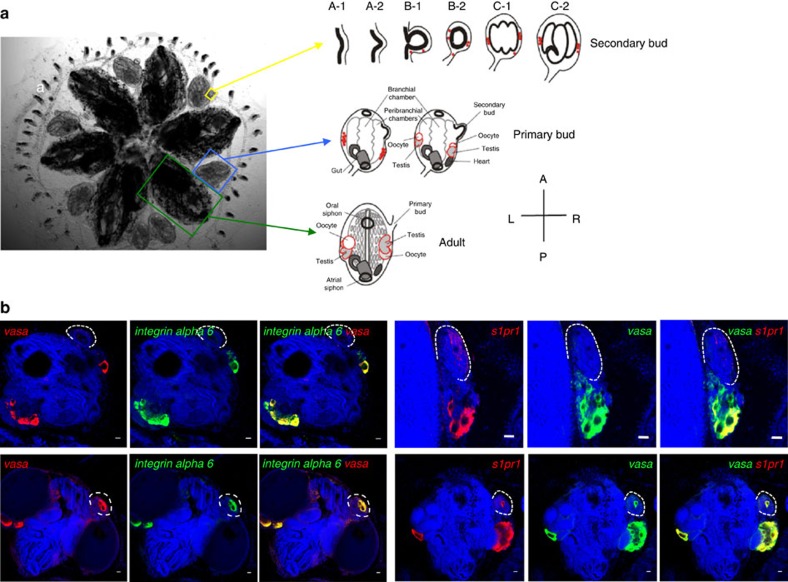
*Botryllus Schlosseri* morphology, gonad formation and expression of *integrin alpha-6* and *s1pr1* in vasa-positive cells. (**a**) Ventral view of a colony of individual adult animals (green boxes and arrows), each of which is connected to asexual propagating primary buds (blue boxes and arrows) and secondary buds (yellow boxes and arrows). During the asexual budding process, new buds form as a thickening of the peribranchial epithelium (stage A), which forms a pocket and eventually closes to form a double vesicle (stage B). Buds undergo invaginations and differentiate into all somatic tissues and organs (stage C). Germ cells (red) enter the newly formed secondary buds at stage B, and differentiate into testes and oocytes, as primary buds develop into the adult form. Individual animals are connected by a common extracorporeal vasculature, which ends in terminal projections known as ampullae **a**. (**b**) Representative examples of expression patterns of *integrina alpha-6* and *s1p*r*1* in *vasa*-positive cells by double-labelled fluorescent *in situ* hybridzation (*n*=5). Left panel: All *vasa*-positive (red) germ cell precursors co-express *integrin alpha*-6 (green). Right panel: *S1pr1* (red) is expressed in *vasa*-positive cells (green). Red and green channels are shown individually together with nuclear counterstaining (blue), and merged images show co-expression of both genes (yellow). Top panels represent stages before homing of *vasa*-positive cells into the secondary buds. Note that the weak red signal on the secondary bud at this stage is due to slight trapping of the probe inside the secondary bud, and not true signal for *s1pr1* mRNA. Bottom panels represent stages after homing of *vasa*-positive cells into the secondary buds. Nuclei were counterstained with Hoechst 33342 (blue). For better orientation, secondary buds have been outlined with broken white lines. Scale bars, 20 μm.

ALDH-positive cells, which are enriched for germ cells, were isolated by flow cytometry. By quantitative reverse transcription (RT)–PCR analysis, we found that ALDH-positive cells express high levels of *s1pr1* and *integrin alpha 6* (Supplementary Fig 1a). *Integrin alpha-6* is expressed at 12.1-fold higher levels in ALDH-positive cells with respect to ALDH-negative cells, and *s1pr1* is expressed at 6.6-fold higher levels (Supplementary Fig 1a). *S1pr4* was expressed at very low levels (Supplementary Fig 1a). To investigate the expression of *s1pr1* and *integrin alpha-6* in *vasa*-positive germ cells, we performed double labelled whole-mount fluorescent *in situ* hybridization (FISH) for *s1pr1* and *integrin alpha-6* together with *vasa* (Fig. 1b). Double FISH showed that the germ cell-specific gene *vasa* is expressed together with *integrin alpha-6*, and all *vasa*-positive cells were found to be *integrin alpha-6*-positive (Fig. 1b). *S1pr1* expression was detected in almost all *vasa*-positive cells (Fig. 1b), and was not detected outside of the germ line.

### Integrin alpha-6 is a surface marker of germ cell precursors

**Figure 2:**
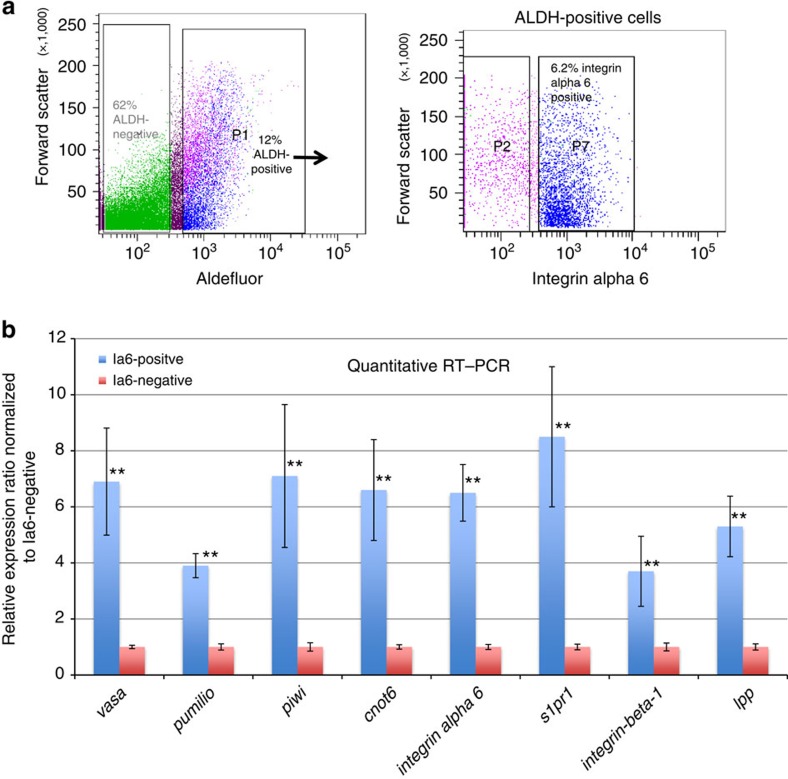
Prospective isolation of germ cell precursors based on expression of aldehyde dehydrogenase and integrin alpha-6 gene expression analysis. **a**) Representative example of fluorescence-activated cell sorting (*n*=20). Aldehyde dehydrogenase (ALDH)-positive and -negative cells were gated based on Aldefluor-fluorescence compared with unstained controls, and 12% of total cells are ADLH-positive. From the ALDH-positive population (arrow), integrin alpha-6-positive (p7) and -negative cells (p2) were gated based on isotype control staining. 6.2% of total cells are ALDH-positive/integrin alpha-6-positve. (**b**) Q-RT-PCR analysis of integrin alpha-6-(Ia6)-positive or -negative cells sorted from the ALDH-positive population. Relative quantification was performed using the 2^-ΔΔCT^-method, with *ef1α* as control gene. Data are expressed as averages of the relative expression ratio (fold change), normalized to Ia6-negative cells. Standard deviations were calculated for each average expression ratio (*n*=3). Statistical analysis was performed using Student’s *t*-test. **P≤0.05 (medium significance compared with Ia6-negative cells).

Previous studies showed that in *Botryllus*, germline precursors can be enriched by flow cytometry based on expression of ALDH, and that ALDH-positive cells functionally reconstituted the germ line of recipients following transplantation, whereas ALDH-negative cells did not^12^. ALDH-positive cells are highly enriched for conserved genes that are known to be important regulators of germ cell fate, and required for GSC maintenance^5^,^16^, such as *vasa*, *piwi*, *pumilio* and *CCR4-NOT complex subunit 6* (*cnot6*; Supplementary Fig 1a). However, only 1 out of 50 cells functionally reconstituted the germ line of a recipient^12^. Based on our *in situ* hybridization data (Fig. 1b), all *vasa*-positive cells also express *integrin alpha-6.* The high level of homology between the human and *Botryllus* integrin alpha-6 at the amino-acid level prompted us to attempt to stain *Botryllus* cells for flow cytometry using an anti-human integrin alpha-6 antibody. In a suspension of cells from *Botryllus* colonies, ALDH-positive cells comprise between 10 and 20% of total cells (Fig. 2a). Around 6% of total cells are double-positive for ALDH and integrin alpha-6 (Fig. 2a). The specificity of the antibody staining was confirmed using an isotype control. We then isolated both integrin alpha-6-positive and -negative cells from the ALDH-positive population, and analysed their gene expression by quantitative RT–PCR (Fig. 2a). *Vasa*, *piwi*, *pumilio* and *cnot6* were significantly enriched in integrin alpha-6-positive (Ia6^+^) cells compared with integrin alpha-6-negative cells (Ia6-), with *vasa, piwi* and *cnot6* being expressed at six- to sevenfold higher levels, and *pumilio* at 3.9-fold higher levels in Ia6-positive cells (Fig. 2b). *Integrin alpha-6, integrin beta-1* and *s1pr1* expression levels were also significantly enriched in Ia6^+^ cells, with *integrin alpha-6* being expressed at 6.5-fold higher levels and *s1pr1* at 8.5-fold higher levels (Fig. 2b). In *Drosophila*, the lipid phosphate phosphatase wunen mediates phospholipid uptake and hydrolysis^8^,^10^. It is expressed at high levels in somatic cells, and regulates directional migration and survival of germ cells, probably by establishing a gradient of an unknown phospholipid substrate. *Wunen* expression is also required in germ cells for their migration and survival (reviewed in ref. 8). The closest mammalian homologues of wunen are lipid phosphate phosphatases (Lpp). *Botryllus* has one Lpp homologue, and it is expressed in Ia6^+^ cells at 5.3-fold higher levels compared with IA6-negative cells (Fig. 2b).

### S1P stimulates migration of germ cells

**Figure 3:**
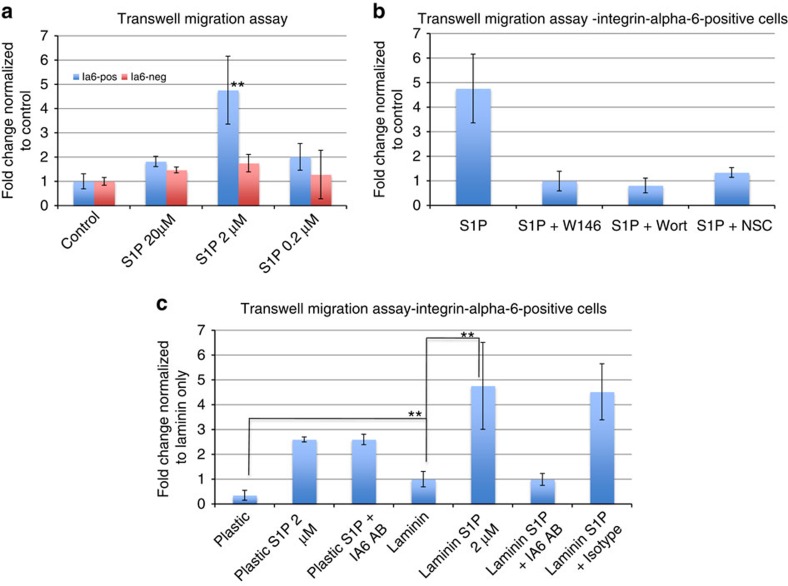
The migration of integrin alpha-6-positive germ cell precursors towards sphingosine-1-phosphate is mediated by the G-protein–coupled receptor S1PR1 and integrin alpha-dependent binding to laminin. (**a**) Migration assay of ALDH-positive cells in response to different concentrations of S1P, as indicated. No stimulant was added to control wells. Sorted cells were added to the upper chamber of a Transwell system coated with laminin, and after 2 h, migrated cells in the lower chamber were counted. Data are expressed as fold changes of numbers of migrated cells, normalized to unstimulated controls (*n*=6). Statistical analysis was performed using Student’s *t*-test. **P≤0.05 (medium significance compared with control). (**b**) Migration assay of ALDH-positive/Integrin-alpha-6-positive cells in the presence of 2 μm S1P with or without inhibitors of S1PR signalling. S1PR1 antagonist W146 (10 μM), PI3K-inhibitor Wortmannin (0.1 μM) or Rac1-inhibitor NSC (100 μM) were added as indicated. Data are expressed as fold changes of numbers of migrated cells, normalized to unstimulated controls. (**c**) Migration assay of ALDH-positive/integrin alpha-6-positive cells on uncoated plastic or laminin-coated Transwells with or without 2 μm S1P. Anti-integrin alpha-6 (5 μg ml^−1^) or isotype control antibody was added as indicated. Data are expressed as fold changes of numbers of migrated cells, normalized to unstimulated controls on laminin. Error bars represent the standard deviation for each average (*n*=6) Statistical analysis was performed using Student’s *t*-test. **P≤0.05 (medium significance).

To directly test whether S1P stimulates chemotaxis of *Botryllus* germ cells, we performed a Transwell migration assay, in which freshly isolated Ia6^+^ or Ia6^−^ cells were placed on top of a Transwell filter. The numbers of cells migrating to different concentrations of S1P in the bottom chamber were quantified and normalized to control wells to which no stimulant was added. Only Ia6^+^ cells responded to S1P with significantly increased migratory activity (*t*-test *P*=0.02), whereas the migration of Ia6^−^ cells was only slightly stimulated by S1P (Fig. 3a). Intriguingly, the migratory response of Ia6^+^ cells was much higher towards 2 μM S1P (4.7-fold) than towards 20 μM S1P (1.8-fold). This is consistent with the concept that cells migrating along a chemotactic gradient will slow their migration once they reach regions with higher concentrations of the chemoattractant, because of receptor desensitization^17^. To test whether this migratory response of germ cells towards S1P is mediated by the *Botryllus* homologue of the vertebrate S1pr-1, which is expressed in *vasa*-positive cells (Fig. 1b), Ia6^+^ cells were exposed to S1P in the presence of the specific S1pr-1-antagonist W146. W146 significantly reduced the migration of Ia6^+^ cells to S1P (*t*-test *P*=0.005), indicating that this migration depends on signalling through S1pr-1 (Fig. 3b). The vertebrate form of the G-protein–coupled S1pr1 activates multiple downstream signalling components, including PI3K and Rac1 (refs 11, 18, 19). We confirmed that the migratory activity induced by S1P in Ia6^+^ cells from *Botryllus* also depends on activation of PI3K and Rac1 downstream of S1pr1, as inhibitors of these signalling molecules (Wortmannin and NSC 23766, respectively) likewise blocked migration to S1P (Fig. 3b).

G-protein–coupled chemokine receptors such as S1pr-1 activate integrins, thereby regulating adhesion, cell polarity and motility. Integrins play important roles in germ cell migration, presumably through their interaction with the extracellular matrix^8^,^20^. Ligand binding by integrin heterodimers results in signal transduction events controlling cell motility. The extracellular matrix protein laminin affects cell migration and polarization, and has been implicated in germ cell migration in mice and flies^8^,^21^,^22^. The integrin heterodimer alpha-6/beta-1 is an important receptor for laminin in neurons, lymphocytes, macrophages, fibroblasts, platelets and other cell types. ALDH-positive/integrin-alpha-6-positive cells express *integrin-beta-1*, leading us to hypothesize that in *Botryllus*, germ cell migration may, at least in part, be mediated by binding to laminin. Laminin alone significantly (*t*-test *P*=0.05) increased the migratory activity of Ia6^+^ cells, and the migratory response to S1P was significantly (*t*-test *P*=0.05) increased on laminin compared with uncoated plastic (Fig. 3c). On laminin, the migratory response to S1P was completely inhibited by an integrin alpha-6-blocking antibody, whereas migration on plastic was unaffected by this antibody (Fig. 3c). These results indicate that integrin alpha-6 is indeed part of a laminin receptor, and that S1pr-1 regulates chemotaxis to S1P by activating Integrin alpha-6.

### *In vivo* expression of *s1pr1* on migrating germ cells

**Figure 4:**
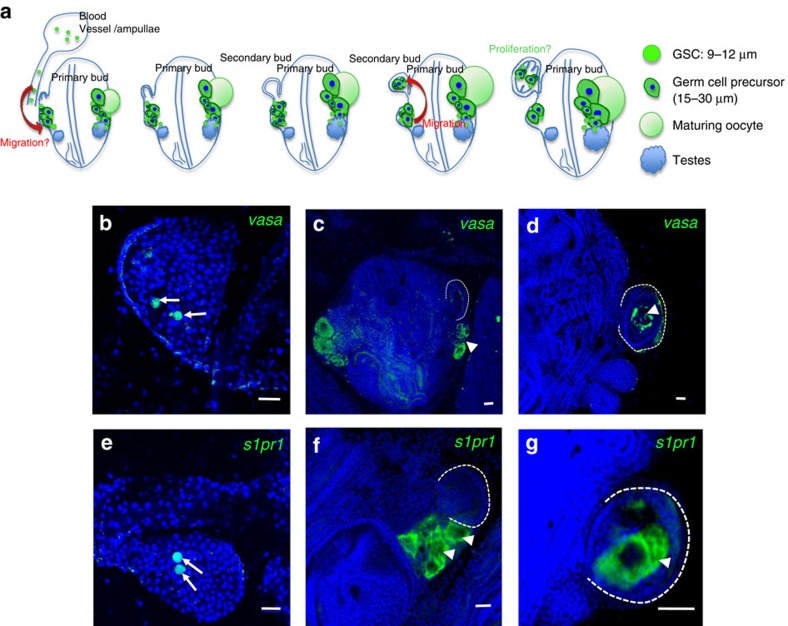
Mobile *vasa*- and *s1p1r*-positive germ cells migrate to secondary buds. (**a**) Schematic representation of the *in situ* hybridization data presented in **b**–**g**. Small, round cells (7–10 μm diameter, green) expressing *vasa* and *s1pr*, are present in the peripheral blood vessels, particularly in ampullae. At early stages of secondary bud development, these small *vasa*/*s1pr1*-positive germline stem cells (GSCs) are also present inside the primary buds, in the vicinity of the developing gonads. We hypothesize that these small GSC that are present in the peripheral blood vessels enter into the primary bud by migration (red arrow), but the exact site and time point of entry is unknown. Larger germ cell precursors (15–30 μm, green), which also express *vasa*, *s1pr1*, likewise cluster around the gonads. When a secondary bud develops and closes to form a double vesicle, germ cell precursors (green) migrate into the secondary bud. As secondary bud development proceeds, more and larger germ cells are visible inside, indicating proliferation and differentiation. (**b**–**g**) Representative images showing expression patterns of *vasa* and *s1p1r* by whole mount fluorescent *in situ* hybridization (green signal) merged with nuclear counterstaining (blue; *n*=10). (**b**) Round, small *vasa* (green)-positive GSC (arrows) inside an ampullae. (**c**) *Vasa*-positive germ cells (arrowhead) next to the developing secondary bud (broken white line). (**d**) *Vasa*-positive germ cells (arrowhead) inside closed double vesicle secondary bud. (**e**) Round, small *s1pr* (green)-positive GSC (arrows) inside an ampullae. (**f**) *s1pr*-positive germ cells (arrowheads) next to the developing secondary bud (broken white line). (**g**) *s1pr*-positive germ cells (arrowhead) inside closed double vesicle secondary bud. Nuclei were counterstained with Hoechst 33342 (blue). Note that small GSC in **b** and **e** have a high nuclear-to-cytoplasmic ratio. The blue signal from the nuclear counterstain overlying the green signal from the FISH appears turquoise. Scale bars, 20 μm.

To characterize the migratory paths of *Botryllus* germ cells *in vivo*, and in particular during the formation of secondary buds, we performed whole-mount FISH for *vasa* and *s1pr1* on colonies in all stages of secondary bud formation. By FISH, *s1pr1* and *vasa* are expressed in small, round cells (7–10 μm in diameter) with the high nuclear-to-cytoplasmic ratio characteristic of primordial germ cells and GSCs (Fig. 4). These cells are present in the peripheral blood vessels, particularly in vascular protrusions known as ampullae, as depicted in the schematic shown in Fig. 4a. We hypothesize that these cells enter into the primary bud from the peripheral blood vessels via an unknown path (Fig. 4a). Figure 4b and e shows FISH images of ampullae containing round, small cells expressing *vasa* and *s1pr1*, respectively (arrows). As shown in the schematic in Fig. 4a, on either side of the developing body, and situated adjacent to and proximal to the newly forming secondary buds, slightly larger germ cell precursors (15–30 μm) are present in addition to those small GSC (Fig. 4c,f, arrowheads, and schematic in Fig. 4a). These precursors also express *vasa* and *s1pr1.* As the newly forming secondary bud closes and forms a double vesicle, as shown in the schematic in Fig. 4a, *vasa*- and *s1pr1*-positive germ cell precursors migrate into the secondary bud (Fig. 4d,g, arrowheads).

### *In vivo* inhibition of S1P signalling blocks germ cell homing

**Figure 5:**
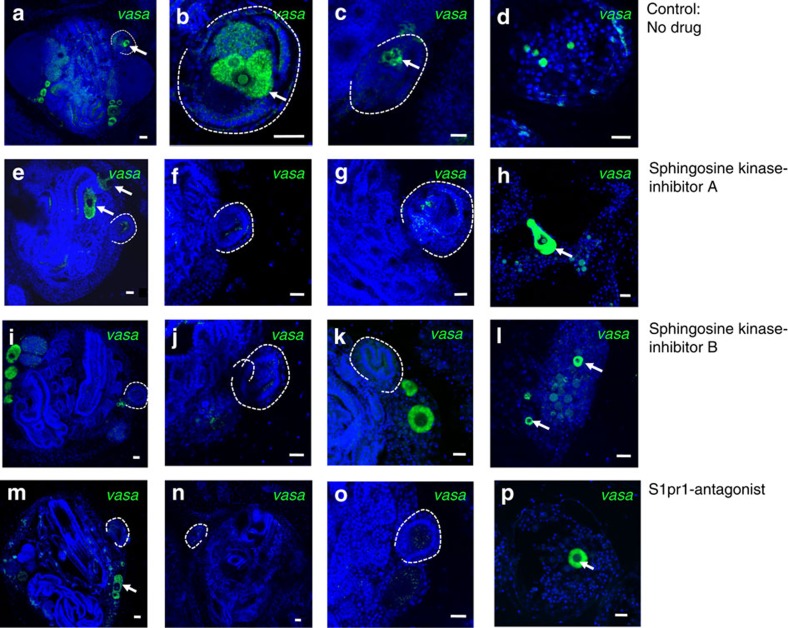
S1P signalling is essential for homing of germ cells into secondary buds. Animals were treated with Sphingosine Kinase inhibitors or S1PR1-antagonist for 3 days, starting at stage A1, and fixed at stage B2, when the secondary bud forms a closed double vesicle (*n*=4). Controls were left untreated. *Vasa*-FISH was performed on fixed animals, and localization of germ cells was analysed by confocal microscopy. (**a–c**) *Vasa*-positive germ cells (green, arrows) homed into the double-vesicle-stage secondary bud (broken line) in a control animal. (**d**) Ampulla of a control animal with *vasa*-positive GSC in the circulation. (**e–h**) In animals treated with the sphingosine kinase inhibitor A (SK1-I), *vasa*-positive germ cells do not home to secondary buds (broken lines), but migrate randomly to other locations in the primary bud (**e**, arrow) and in the blood vessels (**h**, arrow). (**i–l**) In animals treated with the sphingosine kinase inhibitor B (CAS 1177741-83-1), *vasa*-positive germ cells do not home to secondary buds (broken lines). Some larger *vasa*-positive germ cells remain outside of the closed double vesicle (**i** and **k**, arrows), whereas others are found in the blood vessels (**l**). (**m–p**) In animals treated with S1PR1-antagonist, *vasa*-positive germ cells do not home to secondary buds (broken lines). Some larger *vasa*-positive germ cell precursors randomly migrate in the vasculature. Nuclei were counterstained with Hoechst 33342 (blue). Scale bars, 20 μm.

**Figure 6:**
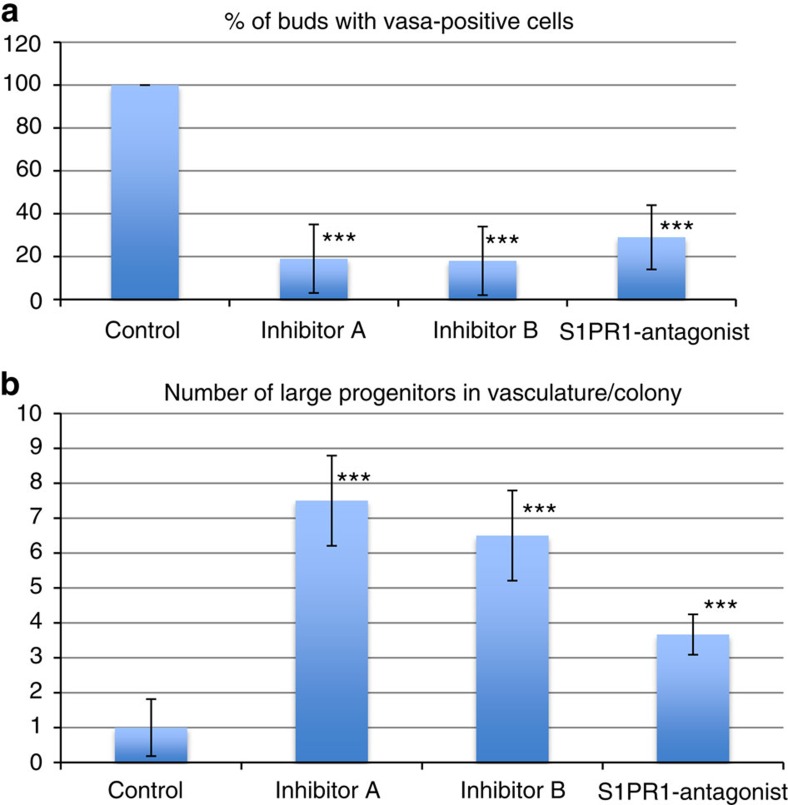
Quantification of migration defects in animals treated with inhibitors of S1P signalling. (**a**) The number of double-vesicle-stage secondary buds containing *vasa*-positive were counted for each condition. Data are expressed as average percentages of buds containing vasa-positive cells, normalized to untreated controls. (**b**) The number of large (>20 μm) *vasa*-positive cells in the vasculature of the colony was counted for each condition. Data are expressed as average number of large *vasa*-positive cells per colony, normalized to untreated controls. Error bars represent the standard deviation for each average (*n*=4). Statistical analysis was performed using Student’s *t*-test. ***P≤0.01 (high significance compared with control).

To test whether S1P regulates homing of germ cell precursors and GSC *in vivo*, animals were treated with small-molecule inhibitors of Sphk1 or an antagonist of S1pr1 (W146). Treatment was started at the stage when the formation of a new secondary bud is in its earliest stage (A1), and barely visible as a thickening of the peribranchial epithelium (Figs 1a,4a left). When the secondary bud reached the double vesicle stage, the localization of germ cells was analysed by FISH for *vasa*. In control animals (untreated), a cluster of *vasa*-positive cells is clearly visible inside the double-vesicle-stage secondary bud (Fig. 5a–c, arrows). In animals treated with inhibitors of Sphk1 or S1pr1 antagonist, double-vesicle-stage secondary buds contain almost no *vasa*-positive germ cells (Fig. 5e–g,i–k,m–o). The number of secondary buds containing any *vasa*-positive cells was significantly reduced in animals treated with Sphk1 inhibitors (Sphk1 inhibitor A *t*-test *P*=0.004, Sphk1 inhibitor B *t*-est *P*=0.01) or S1pr1-antagonist (*t*-test *P*=0.03; Fig. 5m). The absence of *vasa*-positive cells in secondary buds of inhibitor-treated animals could be due to disrupted homing or lack of engraftment and survival in the secondary bud niche, or both. In animals treated with Sphk1 inhibitors or S1pr1 antagonist (W146), *vasa*-positive germ cell precursors were visible in unusual locations inside the primary bud, such as near the endostyle (Fig. 5e, arrows), which indicates that these cells might be migrating randomly due to inhibition of the homing signal. In addition to the small *vasa*-positive GSC that are present in the blood vessels of untreated control animals (Fig. 5a), larger (20–40 μm) *vasa*-positive germ cell precursors were detected inside blood vessels (Fig. 5a,c, arrows) of animals treated with Sphk1 inhibitors or S1pr1 antagonist W146. Compared with control animals, the number of large *vasa*-positive precursors present in the vasculature was significantly increased in drug-treated animals (Fig. 6b). These results demonstrate that in the absence of S1P signalling, germ cell precursors migrate randomly and are not able to home to the secondary buds.

### S1P is generated in the germ cell niche

**Figure 7:**
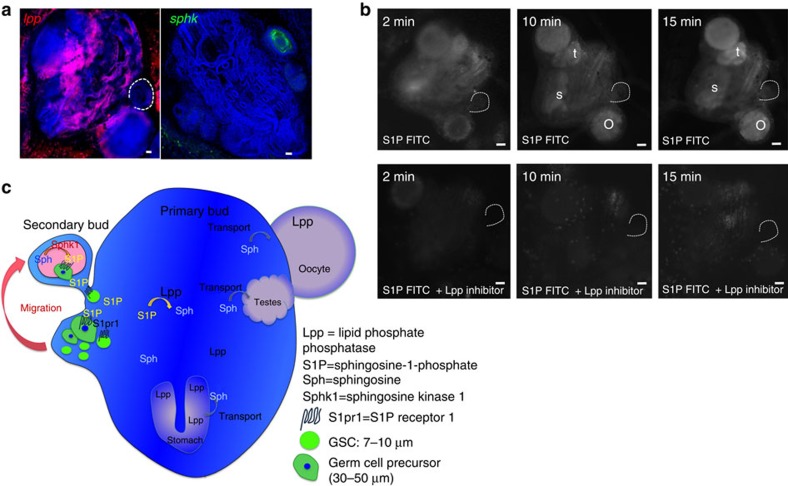
S1P is generated by sphingosine kinase in secondary buds and degraded by LPP in somatic tissues of the primary bud. (**a**) FISH for *sphingosine kinase* (*sphk*, green) and *lipid phosphate phosphatase* (*lpp*, red). *Sphk* is expressed in double-vesicle-stage secondary buds, whereas *lpp* is expressed broadly in somatic tissues of the primary bud. Scale bars, 20 μm. (**b**) Live imaging of fluorescence at different time points after injection of S1P-fluorescein with or without the LPP inhibitor sodium-orthovanadate. After 10 min, S1P-fluorescein accumulates in developing oocytes (o), testes (t) and the stomach (s) of the primary bud. This accumulation is inhibited by inhibition of LPP (*n*=3). Scale bars, 30 μm (**c**) Schematic summary of the *in vivo* data. Vasa-positive germ cells express S1PR1, and migrate towards a gradient of S1P into the germ cell niche in the secondary bud, where S1P is produced by Sphk. In somatic tissues of the primary bud, LPP dephosphorylates S1P to sphingosine, which then gets actively taken up by cells in the developing gonads and the stomach.

The formation of extracellular gradients of S1P depends on the organization of cell types with varying S1P metabolism^23^. Some cell types are specialized metabolically to produce high extracellular levels of S1P, whereas other cell types are metabolically geared towards keeping the extracellular S1P levels low^23^. To assess the mechanism that results in the formation of different amounts of S1P within the double-vesicle-stage secondary buds and somatic tissues outside of the secondary bud, we analysed the expression pattern of *sphk1*. By FISH, *sphk1* expression was detected in developing secondary buds at the time when germ cells migrate, at the double vesicle stage (Fig. 7a). LPPs have been implicated in being the major factor contributing to the formation of changes in S1P levels, as other S1P degrading factors, such as S1P-phosphatase and S1P-lyase, are intracellular enzymes and involved in intracellular metabolism of S1P^23^. In contrast to *sphk1*, *lpp* expression was detected in somatic tissues of the entire primary bud (Fig. 7a, but was absent from the germ cell niche. By FISH, *lpp* expression was not detected in germ cells, even though some expression of *lpp* was detected in ALDH/integrin alpha-6-positve cells by quantitative RT–PCR (Fig. 2b), indicating that expression levels of *lpp* in germ cells are below the limit of detection by FISH.

To functionally assess the role of LPP in the formation of an S1P gradient, we injected fluorescein-labelled S1P into the blood stream of colonies at the double vesicle stage. Fluorescein-S1P diffuses rapidly into the blood vessels and tissues surrounding the injection site (Fig. 7b). After 10 min, the FITC label begins to accumulate in developing testes, oocytes and the stomach of the primary bud, and this accumulation becomes stronger after 15 min, indicating active uptake of the fluorescein label in those tissues (Fig. 7b). These results are in line with reports that dephosphorylation of S1P by Lpp seems to be necessary for uptake of the resulting sphingosine by cells (reviewed in ref. 23). In fact, sphingosine-fluorescein injection resulted in the same pattern of accumulation of fluorescence as S1P-fluorescein (Supplementary figure 1c), suggesting that dephosphorylation of S1P-fluorescein by Lpp leads to uptake of the resulting sphingosine-fluorescein by developing gonads and the stomach. To confirm this hypothesis, we injected S1P-fluorescein together with the phosphatase inhibitor sodium orthovanadate (SOV), which inhibits activity of Lpp^24^. In the presence of SOV, no accumulation of fluorescence was detected, and the injected S1P-fluorescein diffuses rapidly (Fig. 7b).

As summarized in Fig. 7c, our results show that in *Botryllus*, migration and homing of germ cells to newly developing niches depends on signalling of S1P through S1pr1. S1P is generated by Sphk1 in the developing germ cell niche inside the secondary bud. Dephosphorylation and clearance of S1P appear to be mediated by Lpp expression in somatic tissues of the primary bud.

## Discussion

In the present study, we identified a novel role for the phospholipid S1P and the G-protein–coupled receptor S1pr1 in chemotactic migration of germ cells. In addition, we have shown that S1P/S1pr1 induced migration of germ cells depends on integrin alpha-6-mediated binding to laminin, and that integrina alpha-6 is a marker for germ cell precursors in *Botryllus*. When S1P signalling is disrupted, germ cell precursors migrate randomly and are not able to home to the secondary buds. S1P is generated by Sphk1, which is expressed in developing germ cell niches, whereas Lpp mediates dephosphorylation and clearance of S1P in somatic tissues outside of the germ cell niche.

We have demonstrated that in *Botryllus*, an invertebrate chordate, S1P activates a homologue of the vertebrate S1pr1, inducing chemotactic migration of germ cells. This migratory response is dependent on activation of PI3K and Rac1 downstream of the S1P receptor 1, and, ultimately, activation of integrin alpha-6. G-protein–coupled receptors mediate cellular responses to attractive chemokines in a variety of cell types, including germ cells. Tre-1 is a G-protein–coupled receptor of the rhodopsin family, and is important for the migration of primordial germ cells across the posterior midgut in *Drosophila*^8^,^25^. In mice and zebrafish, primordial germ cells express CXCR4, which controls migration to SDF-1 expressed by somatic cells of the developing gonads^8^. The G-protein–coupled S1PR in *Botryllus* is a novel example of this common theme of germ cell chemotaxis, with a previously unknown role in germ cell migration. A role of S1P in germ cell migration has never been shown previously. SphK1/SphK2 double-knockout mice or S1PR1-knockout mice are not viable^26^,^27^, and the role of S1P signalling in germ cell migration in mammals has not been studied to date, but S1P signalling has been well studied for its role in leukocyte migration^11^. Primordial germ cell migration most closely resembles amoeboid migration and the migration of immune cells, which is characterized by individually migrating cells with a broad leading edge, highly dynamic morphology and low adhesiveness^8^.

Lipid signalling pathways are a common theme in the process of germ cell migration that is shared among several species in different phyla (reviewed in ref. 8). In *Drosophila*, the lipid phosphate phosphatases WUN and WUN2, the fly homologues of mammalian LPP’s, are expressed in somatic cells, where they repel migrating germ cells^10^. WUN and WUN2 function by hydrolysing phospholipids and have also been shown to promote the uptake of dephosphorylated lipids. Thus, wunen activity creates a gradient of an unknown phospholipid substrate, which regulates chemotactic migration of germ cells^8^,^28^. Our data demonstrate that a similar mechanism exists in *Botryllus*, where Lpp is involved in degradation of the phospholipid S1P, which regulates migration of germ cells. This suggests a high degree of evolutionary conservation of this mechanism, as tunicates and flies are phylogenetically very distant from each other. Based on our findings in *Botryllus*, it would be interesting to test the role of S1P in germ cell migration in *Drosophila*.

In our study, *lpp* expression was detected on integrin alpha-6-positive cells by quantitative PCR (qPCR), but the levels of expression were too low to be detected by FISH (Figs 2b and 7a). A possible explanation for this is that *lpp* expression might be required for signal termination at the S1P-receptor. S1PR receptor desensitization can occur by receptor internalization^29^, but Pyne *et al*.^18^ proposed an interesting mechanism that involves the dephosphorylation of S1P to sphingosine by LPP1, which is located at the plasma membrane, with an externally oriented catalytic site. Thus, LPP1 may decrease extracellular S1P levels to limit its bioavailability at S1P-receptors. This might require a coordinated mechanism that enables S1P to induce downstream signalling responses before LPP1 is activated to reduce the extracellular concentration of S1P. This could involve an intracellular phosphorylation/dephosphorylation mechanism at cytoplasmic-facing phosphorylation sites of LPP. This mechanism may explain *lpp*-expression on *Botryllus* germ cells (Fig. 2b) and wunen expression on migrating germ cells in *Drosophila*. We will test this possibility in future studies.

In our study, disruption of S1P signalling leads to random migration of germ cells. Therefore, it is likely that other pathways exist that stimulate this random migratory activity of *Botryllus* germ cells. In other animal models, receptor tyrosine kinases often provide such signals, whereas chemoattractants signalling through G-protein–coupled receptors provide directional cues. An example of this is primordial germ cell migration in mice, where SDF1 and CXCR4 control directional migration, and c-kit is required for motility^30^. In future experiments, we will test for the role receptor tyrosine kinases in *Botryllus* germ cell migration.

In conclusion, our data show that germ cells in *Botryllus* express a homologue of the vertebrate G-protein–coupled receptor S1PR1, and that S1P acts as a chemoattractant, directing the homing of germ cells to niches in newly formed asexual progeny. This is the first description of a role of S1P signalling in germ cell chemotaxis.

## Materials and methods

### Animals

*B. schlosseri* colonies used in this study were lab-cultivated strains, spawned from animals collected in Santa Barbara, CA, and cultured in laboratory conditions at 18–20 °C according to the details mentioned in the ref. 31. Animals are reared in 5 l tanks supplemented with food in suspension daily, and food is not limiting. Collections were performed at only one local harbour, the Santa Barbara Harbor (Longitude -119.6887448 and Latitude 34.407), which is owned by the City of Santa Barbara and performed under the authority of the California Department of Fish and Game. These collections did not involve any endangered or protected species. Colonies were developmentally staged according to the details mentioned in the ref. 2.

### Cell sorting

Genetically identical, stage-matched animals were pooled, and a single-cell suspension was generated by mechanical dissociation. Whole animals were minced and passed through 70-μm and 40-μm cell strainers in ice-cold sorting buffer (filtered sea-water with 2% horse serum and 50 mM EDTA). Aldefluor dye (Stem Cell Technologies) was added to cell suspensions at 1/100 dilution, together with Anti-Human/Mouse-CD49f–eFluor450 (Ebioscience, cloneGoH3, 1/100 dilution) and incubated on ice for 30 min and washed with sorting buffer. FACS was performed using a FACSAria (BD Biosciences) cell sorter. Samples were gated as ALDH-positive or -negative based on unstained control fluorescence, and as CD49f-positive or -negative based on isotype control staining (RatIgG2A-isotype-control eFluor450, Ebioscience). Analysis was performed using FACSDiva software (BD Biosciences). Cells were sorted using a 70-μm nozzle and collected into sorting buffer.

### Quantitative RT–PCR

Sorted cells were pelleted at 700 g for 10 min, and RNA was extracted using the Nucleospin RNA XS kit (Macherey Nagel), which included a DNAse treatment step. RNA was reverse transcribed into cDNA using random primers (Life Technologies) and Superscript II Reverse Transcriptase (Life Technologies). Quantitative RT-PCR was performed using a LightCycler 480 II (Roche) and LightCycler DNA Master SYBR Green I detection (Roche) according to the manufacturer’s instructions. The thermocycling profile was 5 min at 95 °C, followed by 45 cycles of 95 °C for 10 s, 60 °C for 10 s. The specificity of each primer pair was determined by BLAST analysis (to human, *Ciona* and *Botryllus* genomes), by melting curve analysis and gel electrophoresis of the PCR product. To control for amplification of genomic DNA, ‘no RT’-controls were used. Primer pairs were analysed for amplification efficiency using calibration dilution curves. All genes included in the analysis had cycle threshold (CT) values of <35. Primer sequences are listed in Supplementary Table 1. Relative gene expression analysis was performed using the 2^-ΔΔCT^ Method. The CT of the target gene was normalized to the CT of the reference gene *elongation factor 1 alpha* (EF1a): Δ*C*_T_=*C*_T (target)_–C_T (EF1α)_. To calculate the normalized expression ratio, the Δ*C*_T_ of the test sample (IA6-positive cells) was first normalized to the Δ*C*_T_ of the calibrator sample (IA6-negative cells): ΔΔ*C*_T_=Δ*C*_T(IA6-positive)_-Δ*C*_T(IA6-negative)._ Second, the expression ratio was calculated: 2^-ΔΔCT^=Normalized expression ratio. The result obtained is the fold increase (or decrease) of the target gene in the test samples relative to IA6-negative cells. Each qPCR was performed at least three times on cells from independent sorting experiments, and each gene was analysed in duplicate in each run. The Δ*C*_T_ between the target gene and EF1alpha was first calculated for each replicate and then averaged across replicates. The average Δ*C*_T_ for each target gene was then used to calculate the ΔΔ*C*_T_ as described above. Data are expressed as averages of the normalized expression ratio (fold change). Standard deviations were calculated for each average normalized expression ratio. Statistical analysis was performed using a paired, two-sided Student’s *t*-test. **P≤0.05.

### *In situ* hybridization

Whole mount *in situ* hybridization was performed as described in ref. 32. Briefly, *B. schlosseri* homologues of genes of interest were identified by tblastn searches of the *B. schlosseri* EST database (http://octopus.obs-vlfr.fr/public/botryllus/blast_botryllus.php) using human or Ciona (when available) protein sequences. Primer pairs were designed to amplify a 500- to 800-bp fragment of each transcript (primer sequences in Supplementary Table 2). PCR was performed with Advantage cDNA Polymerase (Clontech, 639105) and products were cloned into the pGEM-T Easy vector (Promega, A1360). Cloned fragments were sequenced and analysed by Blastx to ensure that the correct sequence was cloned. *In vitro* transcription of antisense probes was performed with SP6 or T7 RNA polymerase (Roche, 10810274001, 10881767001) using either digoxigenin or dinitrophenol labelling. *B. schlosseri* were anaesthetized with 0.02% Tricaine (TCI, T0941) in sea water for 10 min, and then fixed with 4% formaldehyde in 0.5 M NaCl, for 3 h at room temperature. Fixed samples were then dehydrated with methanol and bleached overnight in 6% H_2_O_2_ in methanol under direct light. Samples were rehydrated stepwise into PBST and permeabilized by treatment with 10 mg ml^−1^ proteinase K (Roche, 03115879001) in PBST for 30 min at room temperature. A post-fixation step was performed with 4% formaldehyde in PBST for 20 min at room temperature. Samples were then washed with PBST and incubated in hybridization buffer (65% formamide, 5 × SSC, 1 × Denhardt’s solution, 0.1% Tween-20, 5 mg ml^−1^ torula yeast RNA, 50 mg ml^−1^ heparin) without probe for 4–6 h at 58–65 °C, followed by overnight incubation with digoxigenin-labelled riboprobes diluted in hybridization buffer at 58–65 °C. Control samples were incubated with sense probes. Following hybridization, unbound probes were washed away by a series of low-salt washes and samples were incubated in blocking solution (PBST, 5% heat-inactivated horse serum, 2 mg ml^−1^ bovine serum albumin) for 4 h at room temperature. Probes were detected using horseradish peroxidase-conjugated anti-digoxigenin antibody (Roche, 11207733910) at a dilution of 1/1,000 or horseradish peroxidase-conjugated anti-dinitrophenol antibody (Perkin Elmer, FP1129) at a dilution of 1/200. After incubation with antibody, the samples were washed extensively in PBST+2 mg ml^−1^ bovine serum albumin and transferred to PBS. Digoxigenin-labelled probes were then detected by fluorophore deposition using the TSA Plus System (Perkin Elmer, NEL753001KT). Nuclei were stained with Hoechst 33342 (Life Technologies). Imaging of labelled samples was performed using an Olympus FLV1000S Spectral Laser Scanning Confocal.

### Transwell migration assay

Transwell filters with 8 μm pore size inserted in a 24-well plate (Corning) were coated with laminin over night at 4 °C and briefly air dried before adding 50,000 sorted cells, resuspended in 100 μl filtered sea water with 10% DMEM and 1% FBS. The bottom of the well contained filtered sea water with 10% DMEM/1% FBS. S1P (0.2–20 μM), 10 μM S1PR-1-inhibitor W146, 0.1 μM PI3K-inhibitor Wortmannin, 100 μM Rac1-inhibitor NSC 23766 (all from Tocris), 5 μg ml^−1^ anti-Integrin alpha-6 (clone GoH3, Ebioscience) or isotype control antibody were added to the bottom chamber where applicable. After 2 h incubation at room temperature, nuclei were stained with Hoechst 33342 and counted in images taken at three random locations of the bottom well (at × 100 magnification) using the ‘cell counter’ plugin in FIJI software. All assays were performed in triplicates with cells from three independent sorts. Statistical analysis was performed using a paired, two-sided Student’s *t*-test.

### Small-molecule inhibitor treatment

Animals were incubated in 30 ml of sea water containing 1 μM Sphingosine Kinase Inhibitor (A) SKI-I (Abcam), 14 μM Sphingosine Kinase Inhibitor (B) CAS 1177741-83-1 (Millipore 567731) or 10 μM S1PR1 antagonist W146 (Tocris). Sea water containing drugs was replaced and fresh food (algae) was added daily. Controls were incubated in sea water without inhibitors with vehicle 0.1% ethanol or 0.001% dimethylsulphoxide. After 4 days, animals were fixed and analysed by *in situ* hybridization as described above. For each treatment, two genetically identical colonies were treated simultaneously, and the experiment was performed three times. Buds containing *vasa*-positive cells and the number of *vasa*-positive cells in the vasculature were counted on each treated and untreated colony under an epifluoresence microscope. Data are expressed as averages from three experiments, and statistical analysis was performed using a paired, two-sided Student’s *t*-test.

### S1P-fluorescein live imaging

Colonies were microinjected into the blood stream with 1 μl of 0.1 mg ml^−1^ S1P-fluorescein in sea water, with or without 10 mM SOV. Colonies were placed under sea water on top of an inverted epifluorescence microscope (Olympus) equipped with a cooled camera. Images of green fluorescence were taken at 2 min after injection, and every 5 min thereafter.

## Additional information

**How to cite this article:** Kassmer, S. H. *et al*. Migration of germline progenitor cells is directed by sphingosine-1-phosphate signalling in a basal chordate. *Nat. Commun.* 6:8565 doi: 10.1038/ncomms9565 (2015).

## Supplementary Material

Supplementary Information Supplementary Figure 1 and Supplementary Tables 1-2
