# Supplementary material for: Migration of germline progenitor cells is directed by sphingosine-1-phosphate signalling in a basal chordate
Source: Nat Commun. 2015 Oct 12;6:8565. doi: 10.1038/ncomms9565 (PMC4606877; doi:10.1038/ncomms9565)
Supplement: Supplementary Information — Supplementary Figure 1 and Supplementary Tables 1-2 [file ncomms9565-s1.pdf]

Supplementary Figure 1

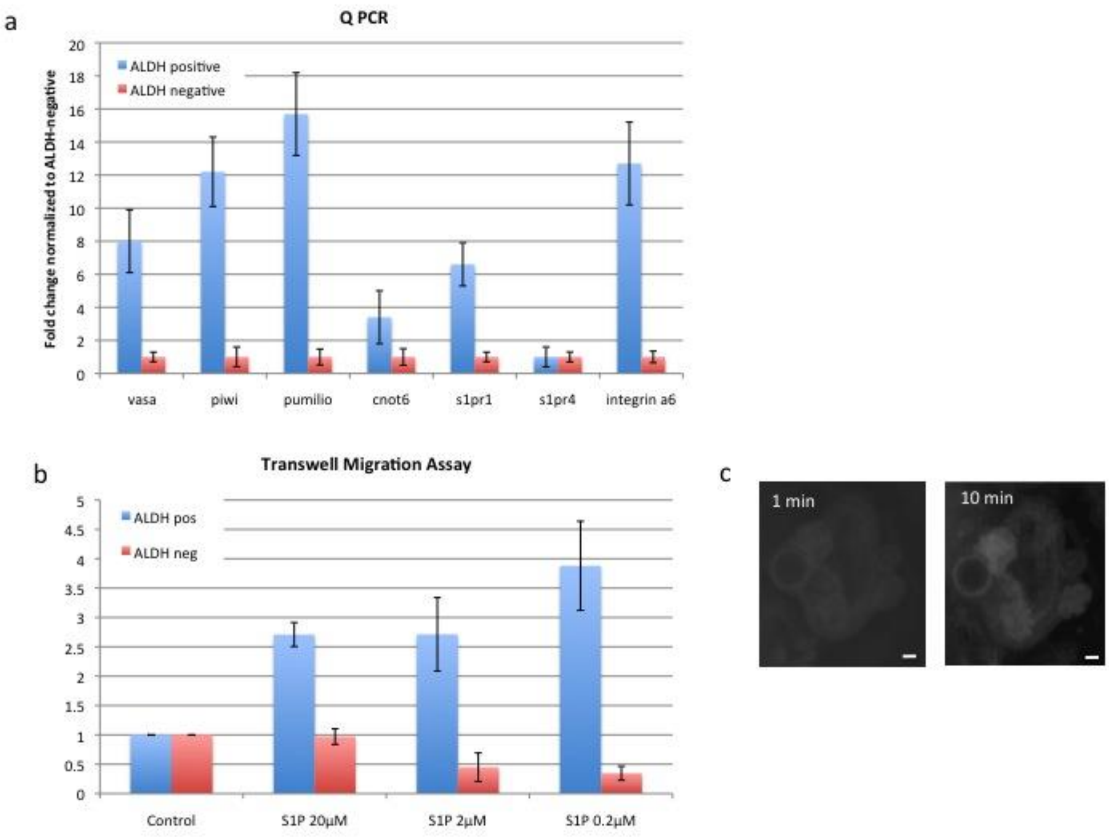

Supplementary Figure 1: **RT-PCR and migration assay of ADLH-positive cells, Sphingosine-Fluorescein live imaging. a)** Q-RT-PCR analysis of sorted ALDH-positive and ALDH-negative cells. Relative quantification was performed using the $2^{-\Delta\Delta CT}$-method, with *ef1α* as control gene. Data are expressed as averages of the expression ratio (fold change), normalized to ALDH-negative cells. Standard deviations were calculated for each average expression ratio. **b)** Transwell migration assay of sorted ALDH-positive and ALDH-negative cells in response to decreasing concentrations of S1P. No stimulant was added to control wells. Sorted cells were added to the upper chamber of a transwell system, and after 2 h, migrated cells in the lower chamber were counted. Data are expressed as fold changes of numbers of migrated cells, normalized to controls. Error bars represent the standard deviation for each average (n=4).**c)** Control for experiment shown in Figure 7 B. Live imaging of fluorescence intensity after injection of Sphingosine-Fluorescein into the blood stream. After 10 minutes, Sphingosine-Fluorescein accumulates in developing oocytes, testes and the stomach of the primary bud. Scale bars = 30μm.

Supplementary Table 1: qPCR primers

ef1a –Forward	CGTGGTCATTGGCCACGTAGA	Efficiency
ef1a-Reverse	ATGAAATCACGATGACCGGGA	100%
Vasa-forward	GGCGGATTTAGCGATGATGAG	101%
Vasa-reverse	TTCCCCCATAGCGACTGTTAGAC	
Piwi	GACAACGAGCTACGCAACTGC	91%
Piwi	CTCACGTTGAACGCCAGACTT	
Pumilio	GTCCATGTACGGTTCTGCCA	108%
pumilio	TTCGGGAAACGGTTGTTCCT	
Cnot6	CTACGACGACACTGAGCAGG	107%
Cnot6	TCGGTGGGGGCATCCTAATA	
S1pr1 Forward	TTACTCCATCAGGATGCCGC	93%
S1pr reverse	TTCTGCGAGATTTCCGCCTT	
Integrin alpha 6	ACTTCCGGCACGAACAAGAT	110%
Integrin alpha 6	GTACAACAGGGTAACCGGGG	
Integrin beta-1	TTCGTGTTGATACGGTCGCA	105%
Integrin-beta 1	GCACCAATCCAAACACGGAC	

Efficiency: Primer pairs were analyzed for qPCR amplification efficiency using calibration dilution curves. Amplification efficiency is expressed as percentage.

Supplementary Table 2: Cloning primers

		Product length
Vasa-forward	AGGCACTATGATTCAGCCTGTG	976
Vasa-reverse	ATCATAATCACCCGTCTCGCG	
S1pr1 Forward	GTCGCGTGTTTGATAGCGTC	
S1pr1 reverse	TTCTGCGAGATTTCCGCCTT	979
Integrin-alpha-6-forward	TCGGGGTTTTGTAGGTCGTG	657
Integrin-alpha-6-reverse	GCGATAACGATCGGACTGGT	
